# Suppnonsense-mediated decay-linked mutations in SARS-CoV-2 and their association with COVID-19 disease severity

**DOI:** 10.1186/s12879-025-11609-8

**Published:** 2025-09-16

**Authors:** Omnia M. Abdel-Haseb, Salwa Sabet, Wael A. Hassan, Ahmed Abd El-Raouf, Usama Bakry, Mohamed Gomaa Seadawy, Ahmed F. Gad, Mohamed Abdel-Salam Elgohary, Nashwa El-Khazragy

**Affiliations:** 1 Egypt Center for Research and Regenerative Medicine (ECRRM), Cairo, 11599 Egypt; 2 Department of Zoology, Faculty of Science, Cairo University, Cairo, 12613 Egypt; 3 Biodefense Center for Infectious and Emerging Diseases, Ministry of Defense, Cairo, 11775 Egypt; 4 Department of Clinical Pathology-Hematology and AinShams Medical Research Institute (MASRI), Faculty of Medicine, Ain Shams University, Cairo, 11566 Egypt

**Keywords:** COVID-19, Nonsense-mediated decay, SARS-CoV-2, Variants, Whole-genome sequencing

## Abstract

**Background:**

Nonsense-mediated decay (NMD) is a cellular mechanism that degrades mRNAs with premature termination codons (PTCs), preventing the production of truncated, potentially harmful proteins. While its role in viral infections is increasingly recognized, the relationship between NMD-linked mutations in SARS-CoV-2 and COVID-19 severity remains poorly understood.

**Objective:**

To investigate the presence of SARS-CoV-2 nonsense mutations predicted to trigger NMD and assess their association with clinical disease severity and viral genomic.

**Methods:**

We conducted whole-genome sequencing on samples from 129 hospitalized COVID-19 patients. A panel of 21 nonsense mutations predicted to activate the NMD pathway was identified and analyzed. Statistical correlations with clinical severity were assessed, including multivariate analysis. Receiver Operating Characteristic (ROC) curves and interdependency analysis of mutation combinations were also performed.

**Results:**

Five NMD-associated mutations (Variants 5, 6, 7, 9, and 15) showed significant associations with mild disease. These mutations, located in key SARS-CoV-2 genes, include Variant 5 (g.C5575G, T, synonymous substitution in ORF1ab), Variant 6 (g.T5653G, substitution in ORF1ab), Variant 7 (g.G6094GGACAGACTTT/GCCTACACGACGCTAATC, insertion in spike protein), Variant 9 (g.G6446GAATGA, insertion in spike protein), and Variant 15 (g.T10968TATATTGA, insertion in N protein). In the host-adjusted multivariable model, the presence of at least one NMD-inducing mutation was an independent protective factor (OR = 0.34, 95% CI: 0.16–0.72, *p* = 0.005). However, after adjusting for SARS-CoV-2 lineage, this association was attenuated (OR = 0.67, 95% CI: 0.03–13.84, *p* = 0.793). In the lineage-only model, Omicron infection showed higher odds of severe disease compared to Delta (OR = 1.91, 95% CI: 0.77–4.77, *p* = 0.164). ROC analysis indicated limited predictive value for individual variants (AUC = 0.37–0.48), but specific combinations, such as Variants 5 and 7, markedly reduced severe case incidence (92.9–4.8%, *p* = 0.0001).

**Conclusion:**

NMD-inducing nonsense mutations were associated with reduced COVID-19 severity in host-adjusted analyses, but this effect diminished after accounting for viral lineage, suggesting that variant distribution, particularly Omicron may influence these associations. Integrating viral genomic background with host and clinical data may enhance risk prediction and inform antiviral strategies.

**Supplementary Information:**

The online version contains supplementary material available at 10.1186/s12879-025-11609-8.

## Introduction

The coronavirus disease 2019 (COVID-19), caused by SARS-CoV-2, has been one of the most impactful global health crises in modern history [1, 2]. Since its emergence in late 2019, the virus has infected over 700 million individuals and led to more than 7 million deaths worldwide [3, 4]. While many patients experience mild to moderate illness, others develop severe complications such as acute respiratory distress syndrome (ARDS), multi-organ failure, and death [5, 6]. This wide variability in clinical outcomes has prompted intense research into the virological and host-related factors that influence disease severity.

One emerging area of interest is the role of *nonsense-mediated decay (NMD)* in viral infections, including COVID-19 [7, 8]. NMD is a conserved cellular surveillance mechanism that degrades messenger RNA (mRNA) transcripts containing *premature termination codons (PTCs)* [9]. This process protects cells from producing truncated, potentially harmful proteins [10]. Beyond its housekeeping role, NMD also regulates the expression of many normal genes and can influence immune responses, stress reactions, and viral replication [11, 12].

Viruses, including SARS-CoV-2, can interact with the host NMD pathway in complex ways [10]. Some viral RNAs may be targeted by NMD, reducing their expression and limiting replication [11, 13]. However, viruses can also evolve to evade or suppress NMD, allowing even defective transcripts to persist and potentially confer advantages such as immune evasion or altered pathogenesis [14]. Therefore, mutations in the viral genome that introduce PTCs or affect RNA splicing, collectively termed NMD-inducing or NMD-associated mutations, may play a role in modulating the clinical course of infection [15]. 

Recent sequencing studies have revealed the presence of NMD-associated mutations across various SARS-CoV-2 lineages, including Variants of Concern (VOCs), such as Alpha, Delta, and Omicron [16]. The frequency and distribution of these NMD-related mutations differ between variants, possibly reflecting evolutionary pressures and host interactions [17]. These mutations, which can introduce premature termination codons (PTCs) or affect RNA splicing, may influence viral gene expression and pathogenicity [18]. It has been demonstrated that the Delta variant, associated with more severe disease, has shown a higher frequency of certain NMD-associated mutations [19], whereas the Omicron variant, despite its high transmissibility, presents a more complex mutational profile with varying impacts on severity [20].

Importantly, the relationship between NMD-associated mutations and COVID-19 disease severity remains poorly understood. On one hand, NMD-triggered degradation of essential viral transcripts may reduce viral load and attenuate disease [7]. On the other hand, mutations that allow the virus to escape NMD could support ongoing replication and immune evasion, thereby worsening clinical outcomes [21, 22]. Additionally, host genetic differences in the efficiency of NMD machinery may further influence how these mutations affect disease progression [23]. 

Preliminary studies suggest that the presence of NMD-inducing mutations in SARS-CoV-2 may correlate with increased disease severity in some cases [24, 25], potentially through enhanced inflammatory responses or prolonged viral persistence [26, 27]. However, systematic investigations linking these mutations with clinical data across large patient populations are limited.

This study aims to explore the association between NMD-inducing mutations in the SARS-CoV-2 genome and the severity of COVID-19 in infected individuals. By analyzing the genomic sequences of viral isolates from patients with varying clinical presentations, we will assess the frequency, distribution, and functional impact of these mutations. Understanding how viral mutations interact with host RNA surveillance mechanisms such as NMD can provide deeper insights into the molecular underpinnings of COVID-19 pathogenesis. This knowledge may help identify novel biomarkers for disease severity, guide therapeutic development, and inform public health responses to emerging variants.

## Methods

### Patient recruitment and clinical categorization

This study was designed as a retrospective observational cohort study conducted between December 2021 and December 2022. It involved the collection and analysis of clinical and genomic data from 126 patients with RT-PCR–confirmed SARS-CoV-2 infection who were admitted to military hospitals. The objective was to investigate the association between viral NMD-associated mutations and the clinical severity of COVID-19. Ethical approval was obtained from the ***Egypt ***Center For Research and Regenerative Medicine (1/11-2021), and written informed consent was obtained from all participants or their legal guardians. Eligible participants were adults aged 18 years or older who had tested positive for SARS-CoV-2 by real-time reverse transcription polymerase chain reaction (RT-PCR) and were admitted to one of the participating hospitals. Patients were clinically categorized into three severity groups based on the ***World Health Organization*** (WHO) guidelines and institutional criteria: mild (symptomatic without radiologic or clinical signs of pneumonia), moderate (radiographic evidence of pneumonia without need for oxygen supplementation), and severe (requiring supplemental oxygen, intensive care unit admission, or mechanical ventilation). Patients were recruited from a single outbreak during a defined time, ensuring a homogeneous cohort, and statistical analyses were adjusted for comorbidities and age confounders to minimize bias.

Exclusion criteria included the absence of confirmatory RT-PCR results, incomplete clinical records, prior receipt of antiviral therapy before hospital admission, immunocompromised status such as active cancer or organ transplantation, and patient refusal or withdrawal of consent. Clinical and demographic data, including age, sex, comorbidities, date of symptom onset, hospital stay duration, and clinical outcomes, were collected using a standardized case report form completed by trained study staff from patient interviews and electronic medical records.

### Sample collection

Nasopharyngeal (NP) swab specimens were collected from all patients for virological analysis. Swabs were obtained using sterile flocked swabs by trained healthcare professionals following WHO-recommended procedures. The swab was gently inserted through one nostril to reach the posterior nasopharynx, rotated to absorb secretions, and then immediately placed into 3 mL of sterile viral transport medium (VTM).

Specimens were transported to the central virology laboratory within two hours of collection using cold-chain procedures at 2–8 °C. In cases where transport exceeded two hours, samples were stored at 2 °C and shipped under temperature-controlled conditions. Upon arrival, samples were handled in a Class II biosafety cabinet in a Biosafety Level 2 Plus facility. Each sample was assigned a unique barcode and processed according to standard laboratory protocols.

Aliquots were prepared for RNA extraction, RT-PCR confirmation, and whole-genome sequencing. The remaining sample material was stored at − 80 °C for future analyses. All laboratory personnel wore appropriate personal protective equipment (PPE), and strict biosafety and quality control protocols were followed throughout the process to ensure sample integrity and prevent contamination.

Viral genome sequences were obtained from nasopharyngeal swabs collected upon patient admission, followed by RNA extraction and whole-genome sequencing using standard protocols. Sequences were processed and aligned to the reference SARS-CoV-2 genome using bioinformatics tools to identify mutations.

### RNA extraction and sequencing

Viral RNA was extracted from nasopharyngeal swab samples using the QIAamp Viral RNA Mini Kit (***QIAGEN***,*** Hilden***,*** Germany***) according to the manufacturer’s instructions. All extraction steps were performed under sterile conditions in a Class II biosafety cabinet within a Biosafety Level 2 Plus (BSL-2+) facility. Extracted RNA samples were quantified using the Qubit Fluorometer (***Thermo Fisher Scientific***,*** USA***) to ensure sufficient concentration and quality for downstream sequencing applications [28].

### Whole genome sequencing (WGS) of SARS-CoV-2

Whole genome sequencing of SARS-CoV-2 was conducted using the Illumina iSeq 100 platform to generate high-quality viral genomic data for mutation analysis. Following RNA extraction and quantification, complementary DNA (cDNA) synthesis and targeted amplification of the SARS-CoV-2 genome were performed using a tiled amplicon-based approach optimized for high genome coverage. Library preparation was conducted according to the manufacturer’s protocol using the iSeq 100 i1 Reagent v2 (300-cycle) kit (Illumina, PN 20021535), which allows for paired-end sequencing with 2 × 150 bp reads.

Amplicon libraries were prepared by reverse transcription of extracted viral RNA, followed by multiplex PCR using SARS-CoV-2 specific primers designed to amplify overlapping regions across the entire viral genome. After amplification, libraries were cleaned using magnetic bead-based purification and indexed through ligation with unique dual indices to allow for multiplexing of multiple samples per run. The quality and fragment size distribution of the libraries were verified using an Agilent Bioanalyzer or Qubit fluorometric analysis.

The pooled libraries were denatured, diluted, and loaded onto the Illumina iSeq 100 System according to the manufacturer’s instructions. Sequencing runs were monitored in real time to ensure optimal performance metrics, including cluster density, base quality scores, and Q30 values. Each sequencing batch included a no-template control and a positive SARS-CoV-2 control to validate run performance and detect potential contamination [29].

### Data analysis & bioinformatics

#### Raw reads processing and mapping

Raw sequencing reads were demultiplexed and converted to FASTQ format (Version 0.11.9) and MultiQC (Version 1.13) using Illumina’s bcl2fastq software. Quality control checks were performed using FastQC, and adapter sequences and low-quality bases were trimmed using Trimmomatic [30]. High-quality reads were aligned to the SARS-CoV-2 reference genome (NC_045512.2) using the Burrows-Wheeler Aligner (BWA-MEM) (Version 0.7.17-r1188) algorithm. PCR duplicates were marked and removed using Picard Tools, and alignment statistics were reviewed to ensure adequate coverage (> 95% of the genome, with an average depth of ≥ 100×) [31].

#### Variant detection

Variant calling was performed using the iVar pipeline in conjunction with SAMtools (Version 1.17) [32], followed by refinement and confirmation using the Genome Analysis Toolkit (GATK) HaplotypeCaller [33]. Variants were filtered to exclude sequencing artifacts, low-quality calls (Phred score < 30), and regions with coverage < 10×. A consensus genome sequence for each sample was generated based on the high-confidence variants.

Variants were functionally annotated using SnpEff with a custom SARS-CoV-2 database to identify synonymous, non-synonymous, frameshift, and nonsense mutations [34]. We focused specifically on identifying NMD-associated mutations, defined as:

1. Nonsense mutations introduce premature termination codons (PTCs) in protein-coding regions.
2. Frameshift insertions or deletions (indels) are predicted to introduce PTCs downstream.
3. Splice site mutations located within ± 2 base pairs of canonical splice donor or acceptor sites.

To assess the likelihood of NMD targeting, mutations were further analyzed in the context of transcript structure. Specifically, premature stop codons located > 50–55 nucleotides upstream of the final exon-exon junction were flagged as potential NMD-triggering events based on established criteria for NMD recognition in eukaryotic cells. These predictions were cross-referenced with published SARS-CoV-2 transcriptome models and viral open reading frame (ORF) annotations.

All identified NMD-associated mutations were cataloged by genomic position, variant type, affected gene or ORF, and patient severity classification (mild, moderate, or severe). Frequencies were compared across clinical groups to explore potential correlations between NMD mutation burden and disease severity.

Where applicable, mutational profiles were also mapped to known Variants of Concern (VOCs) using lineage assignment tools such as Pangolin and Nextclade, and results were validated against publicly available datasets (GISAID and CoV-GLUE) [35].

Finally, Spearman correlation coefficient analysis between selected mutations was done using the “cor” function in R (Version 4.2), and the sample clustering was performed using the “hclust” function in R (Version 4.2).

#### Viral lineage determination

Whole-genome SARS-CoV-2 sequences from all study participants were analyzed to assign Pango lineages using pangolin v4.3 with the latest pangoLEARN model (accessed July 2025). WHO-recognized variant classifications were mapped from Pango lineages, resulting in four lineage categories in our dataset: Delta (B.1.617.2 and sublineages), Alpha (B.1.1.7 and sublineages), Omicron (BA., XBB., and sublineages), and VOIs/VOCs (other) (e.g., C.36.3, C.36, B.1.351). The results were validated against publicly available datasets (GISAID and CoV-GLUE) [35].

### Sample size calculation

The sample size for this study was determined using power analysis to detect a significant association between the presence of NMD-associated mutations and COVID-19 disease severity. The calculation was based on a two-sided Chi-square test for comparing proportions between groups, with a significance level (α) of 0.05 and a power (1–β) of 80%. An effect size (Cohen’s w) of 0.25 was assumed, representing a medium effect based on preliminary literature and pilot data. The minimum required sample size was calculated to be 120 participants. To account for a potential 5% rate of data exclusion due to poor sequencing quality or incomplete clinical data, the target enrollment was increased slightly. As a result, 129 patients were recruited between December 2021 and December 2022.

The sample size calculation was performed using G*Power version 3.1.9.7 (Heinrich Heine University Düsseldorf, Germany), employing the Chi-square test family for goodness-of-fit (contingency tables). This sample size was deemed adequate to detect statistically significant differences in the frequency of NMD-associated mutations across clinical severity groups (mild, moderate, and severe) with sufficient precision and reliability.

### Statistical analysis

Statistical analyses were performed using SPSS version 26.0 (IBM Corp., Armonk, NY) and GraphPad Prism version 9.0 (GraphPad Software, San Diego, CA). Patient demographic and clinical characteristics were summarized using descriptive statistics. Continuous variables were presented as means ± standard deviations (SD) or medians with interquartile ranges (IQR), depending on data distribution assessed by the Shapiro–Wilk test. Categorical variables were expressed as counts and percentages.

To evaluate the association between NMD-associated mutations and COVID-19 disease severity, patients were grouped into three severity categories (mild, moderate, and severe). The frequency of NMD-inducing mutations was compared across severity groups using the Chi-square test or Fisher’s exact test, as appropriate. Continuous variables such as age, viral load (Ct values), and number of NMD-associated mutations per genome were compared between groups using one-way ANOVA for normally distributed data or the Kruskal–Wallis test for non-parametric data.

We conducted multivariable logistic regression to evaluate the association between NMD-linked mutations, viral lineage, and COVID-19 severity, adjusting for host factors (age, sex, comorbidities). Analyses were conducted in three steps: (1) NMD-positive vs. NMD-negative model, Host-adjusted model assessing the association between NMD status and severity, without including viral lineage, (2) Viral lineage model, Model with WHO variant (Delta as reference category) as the primary exposure, adjusting for host factors, to assess association between lineage and severity, and (3) Lineage-adjusted NMD model, Model including both NMD status and WHO variant (Delta as reference) to assess the independent association between NMD status and severity after accounting for viral lineage.

Odds ratios (ORs), 95% confidence intervals (CIs), and p-values were calculated. Models with insufficient exposure variation (e.g., where all patients in a lineage shared the same NMD status) were noted as not estimable (NE). A **p-value < 0.05** was considered statistically significant.

Receiver operating characteristic (ROC) curve analysis was also performed to evaluate the predictive value of the NMD mutation burden for distinguishing severe from non-severe cases. The area under the curve (AUC) was used to quantify diagnostic performance.

Where relevant, correlations between the number of NMD-associated mutations and clinical outcomes, such as length of hospital stay, were assessed using Spearman’s rank correlation coefficient.

## Results

### Variant filtering and identification of NMD-Associated mutations

Whole-genome sequencing was performed on 129 SARS-CoV-2 samples collected from patients admitted to military hospitals. Following quality control and bioinformatics filtering, an initial total of 2,192 variants were identified. Applying depth (Dp ≥ 100) and functional annotation filters, 444 high-confidence variants were retained for further analysis. Among these, 47 loss-of-function mutations were identified, all located within the open reading frame 1ab (ORF1ab) region. Of particular interest, 21 nonsense mutations predicted to activate the nonsense-mediated decay (NMD) mechanism were selected as the focus of this study.

###  Demographic and clinical characteristics

The clinical and demographic distribution of the studied cohort is shown in Table 1. The cohort consisted of 52 females (39.8%) and 77 males (60.2%), with no statistically significant difference in gender distribution across severity levels (*p* = 0.958). Similarly, age stratification showed no significant association with disease severity (*p* > 0.05 for all age groups).

Patients were stratified into three clinical severity categories, “mild, moderate, and severe,” based on chest Computerized Axial Tomography (CT) findings and clinical parameters. The cohort was then divided into two groups based on the presence (positive) or absence (negative) of one or more of the 21 identified NMD-related variants.

Clinical symptoms such as fever, myalgia, sore throat, cough, and dyspnea did not demonstrate statistically significant differences between the NMD variant-positive and variant-negative groups across severity categories (*p* > 0.05 for all symptoms).

**Table 1:** Demographic and clinical data for the studied cohort:

Clinical data	Positivity	*N* (%)	Mild	Moderate	Severe	*P*-value
Gender	Female	52(39.8)	8(36.8)	27(40.3)	17(40.5)	0.958
	Male	77(60.2)	12(63.2)	40(59.7)	25(59.5)	
Age (18–24)	Negative	12(9.4)	4(21.1)	5(7.5)	3(7.1)	0.153
	Positive	116(90.6)	15(78.9)	62(92.5)	39(92.9)	
Age (25–64)	Negative	123(96.1)	17(89.5)	66(98.5)	40(95.2)	0.540
	Positive	5(3.9)	2(10.5)	1(1.5)	2(4.8)	
Age (≥ 65)	Negative	121(94.5)	17(89.5)	63(94)	41(97.6)	0.190
	Positive	7(5.5)	2(10.5)	4(6)	1(2.4)	
Fever ≥ 38 °C	Negative	20(14.8)	4(15.8)	12(63.2)	4(21.1)	0.484
	Positive	109(85.2)	16(14.7)	55(50.5)	38(34.9)	
Muscle pain (Myalgia)	Negative	62(47.7)	13(19.7)	28(45.9)	21(34.4)	0.241
	Positive	67(52.3)	7(10.4)	39(58.2)	21(31.3)	
Sore throat	Negative	69(53.5)	14(20)	33(48)	22(32)	0.218
	Positive	60(46.5)	5(9)	35(58)	20(33)	
Cough	Negative	35(26.6)	6(14.7)	20(58.8)	9(26.5)	0.625
	Positive	94(73.4)	14(14.9)	47(50)	33(35.1)	
Dyspnea	Negative	84(64.8)	16(19.3)	40(47)	28(33.7)	0.106
	Positive	45(35.2)	3(6.7)	28(62.2)	14(31.1)	
Wheezing	Negative	125(97.7)	19(15.2)	64(51.2)	42(33.6)	0.247
	Positive	4(2.3)	0(0)	3(100)	0(0)	
Chest pain	Negative	91(71.1)	17(18.7)	45(49.5)	29(31.9)	0.156
	Positive	38(28.9)	2(5.4)	22(95.5)	14(35.1)	
Headache	Negative	91(71.1)	14(15.4)	45(49.5)	32(35.2)	0.578
	Positive	38(28.9)	6(13.5)	22(59.5)	10(27	
Joint Pain	Negative	104(81.3)	16(15.4)	53(51)	35(33.7)	0.806
	Positive	25(18.8)	3(12.5)	15(58.3)	7(29.2)	
Nausea/Vomiting	Negative	107(83.6)	17(15.9)	55(51.4)	35(32.7)	0.744
	Positive	22(16.4)	2(9.5)	12(57.1)	8(33.3)	
Coma	Negative	127(99.2)	19(15)	66(52)	42(33.1)	0.632
	Positive	2(0.8)	0(0)	2(100)	0(0)	

N (%): N: Number of patients, (%): percentage within infection severity, *P*-value ≤0.05 considered significant.

### Association between NMD-Associated variants and disease severity

**Fig. 1:**
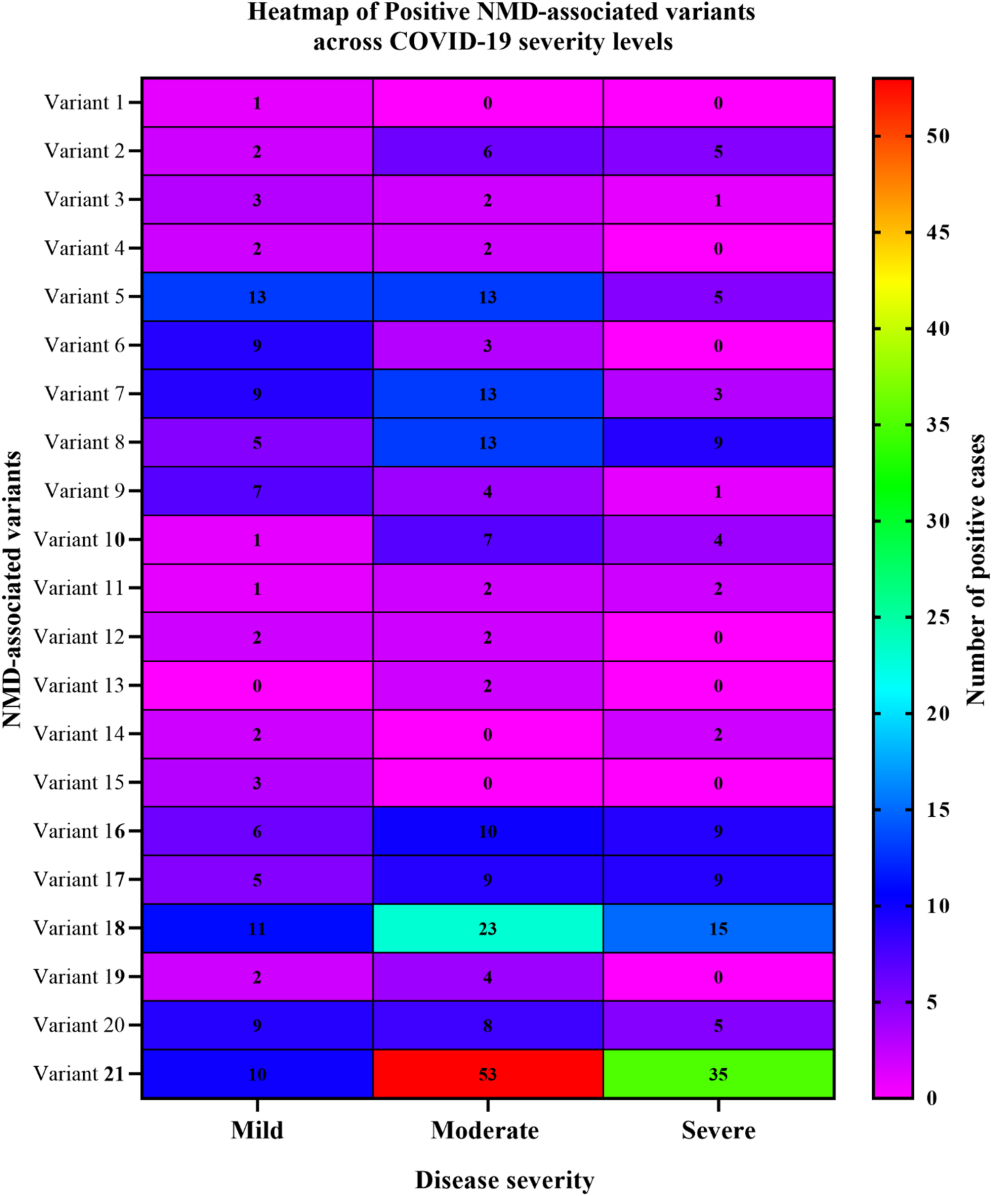
Heatmap of Positive NMD-Associated Variants Across COVID-19 Severity Levels. Heatmap displaying the number of patients who tested positive for each of the 21 identified NMD-associated variants, stratified by COVID-19 severity (mild, moderate, and severe). Each row represents a specific NMD-related nonsense mutation in the SARS-CoV-2 ORF1ab region, and each column indicates a severity level. The color intensity corresponds to the number of patients in each category, with darker shades indicating higher counts

Tables 2 and 3, and Fig. 1. presents the distribution of the 21 NMD-associated variants across patient severity groups and their statistical associations. Among these:

- Variants 5, 6, 7, 9, and 15 showed highly significant associations with disease severity (*p* < 0.001). In each case, patients positive for these variants were more likely to have mild disease, while negative cases showed a higher frequency of severe outcomes, suggesting a potential protective role of these variants.
- Variants 3 and 20 demonstrated moderate significance (*p* ≤ 0.01), indicating a possible association with disease progression, though less pronounced than the highly significant variants.
- Variants 12, 14, and 21 showed mild statistical significance (*p* < 0.05), suggesting weaker but potentially relevant associations.
- The remaining 11 variants (Variants 1, 2, 4, 8, 10, 11, 13, 16, 17, 18, and 19) did not show any statistically significant correlation with disease severity (*p* ≥ 0.05), indicating no detectable relationship in this cohort.

**Fig. 2:**
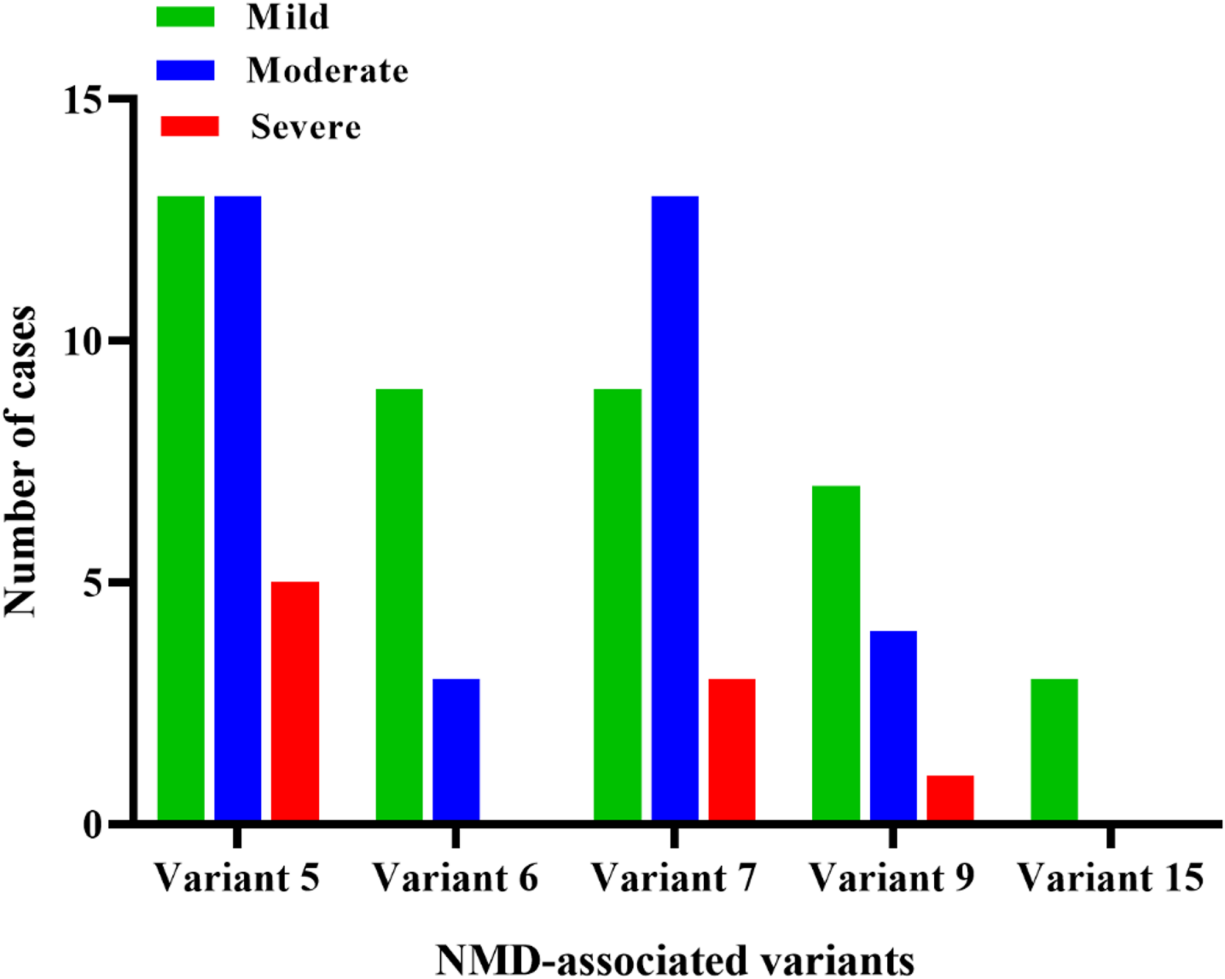
Distribution of COVID-19 Severity Among Positive Cases for Highly Significant NMD-Associated Variants. Bar chart showing the distribution of disease severity (mild, moderate, severe) among patients who tested positive for five NMD-associated SARS-CoV-2 variants that showed high statistical significance in relation to clinical severity (Variants 5, 6, 7, 9, and 15; *p* < 0.001 for each)

These findings highlight a heterogeneous distribution of NMD-associated mutations and their varied influence on clinical severity. Figure 2. Presents that the most significant variants appear to correlate with reduced severity, which means that they have potential protective or neutral effects, supporting the hypothesis that certain NMD-triggering mutations may attenuate viral pathogenicity by interfering with essential viral gene expression and modulating disease outcome.

**Table 2:** Frequencies and distribution of the detected variants in the studied cohort

Variant	Positivity	*N* (%)	Mild	Moderate	Severe	Statistics
Variant 1	Negative	128(99.20	18(94.7)	68(52.8)	42(32.8)	0.056
	Positive	1(0.8)	1(100)	0(0)	0(0)	
Variant 2	Negative	116(89.8)	17(14.8)	62(53)	37(32.2)	0.883
	Positive	13(10.2)	2(15.4)	6(46.2)	5 (38.5)	
Variant 3	Negative	123(96.1)	16(84.2	65(97)	42(99.2)	0.011
	Positive	6(3.9)	3(15.8)	2(3)	1(0.8)	
Variant 4	Negative	125(96.9)	17(89.5)	65(97)	43(100)	0.091
	Positive	4(3.1)	2(10.5)	2(3)	0(0)	
Variant 5	Negative	98(76.6)	7(36.8)	54(80.6)	37(88.1)	0.000
	Positive	31(23.4)	13(63.2)	13(19.4)	5(11.9)	
Variant 6	Negative	117(9.6)	11(52.6)	64(95.5)	42(100)	0.0001
	Positive	12(9.4)	9(47.4)	3(4.5)	0(0)	
Variant 7	Negative	104(80.5)	11(52.6)	54(80.6)	39(92.9)	0.001
	Positive	25(19.5)	9(47.4)	13(19.4)	3(7.1)	
Variant 8	Negative	102(78.9)	14(73.7)	54(80.6)	34(78.6)	0.807
	Positive	27(21.1)	5(26.3)	13(19.4)	9(21.4)	
Variant 9	Negative	117(90.6)	12(63.2)	64(94)	41(97.6)	0.000
	Positive	12(9.4)	7(36.8)	4(6)	1(2.4)	
Variant 10	Negative	117(91.4)	18(94.7)	60(89.6)	39(92.9)	0.714
	Positive	12(8.6)	1(5.3)	7(10.4)	4(2.3)	
Variant 11	Negative	124(96.9)	18(94.7)	65(97)	41(97.6)	0.832
	Positive	5(3.1)	1(5.3)	2(3)	2(4)	
Variant 12	Negative	125(97.7)	17(98.5)	66(98.5)	42(100)	0.034
	Positive	4(2.3)	2(10.5)	2(1.5)	0(0)	
Variant 13	Negative	127(99.2)	19(100)	66(98.5)	42(100)	0.632
	Positive	2(0.8)	0(0)	2(1.8)	0(0)	
Variant 14	Negative	125(97.7)	17(89.5)	67(100)	41(97.4)	0.028
	Positive	4(2.3)	2(10.5)	0(0)	2(2.6)	
Variant 15	Negative	125 (97.7)	16(84.2)	67(100)	42(100)	0.0001
	Positive	4(2.3)	3(15.8)	0(0)	0(0)	
Variant 16	Negative	105(81.3)	14(68.4)	57(85.1)	34(81)	0.259
	Positive	24(18.8)	6(31.6)	10(14.9)	9(19)	
Variant 17	Negative	106(82.8)	14(73.7)	58(86.6)	34(81)	0.391
	Positive	23(17.2)	5(26.3)	9(13.4)	9(19)	
Variant 18	Negative	80(62.5)	8(42.1)	44(65.7)	28(66.7)	0.137
	Positive	49(37.5)	11(57.9)	23(34.4)	15 (33.3)	
Variant 19	Negative	123(96.1)	17(89.5)	64(95.5)	42(100)	0.136
	Positive	6(3.9)	2(10.5)	4(4.5)	0(0)	
Variant 20	Negative	107(83.6)	11(57.9)	59(88.1)	37(88.1)	0.005
	Positive	22(16.4)	9(42.1)	8(11.9)	5(11.9)	
Variant 21	Negative	31(23.4)	9(47.4)	15(20.9)	7(16.7)	0.025
	Positive	98(76.6)	10(52.6)	53(79.1)	35(83.3)	

N (%): N: Number of patients, (%): percentage within infection severity, *P*-value ≤ 0.05 considered significant

**Table 3:** NMD-associated variants

No	Variants	Variants
1	g.C589CTAGT	ins TAGT
2	g.G2654T	Substitution
3	g.C3444CCGGCAGCCCCATCGGTCCTCTCGCATGGAGCTTCTGTGTGGAAACTAA, CCCTACACGGAACGTTCTGAAAAG	insCGGCAGCCCCATCGGTCCTCTCGCATGGAGCTTCTGTGTGGAAACTAA/ins CCTACACGGAACGTTCTGAAAAG
4	g.T3836TGTTAAAAACAGTACAATTCTG	insGTTAAAAACAGTACAATTCTG
5	g.C5575G, T	Substitution/Substitution synonymous substitution
6	g.T5653G	Substitution
7	g.G6094GGACAGACTTT, GCCTACACGACGCTAATC	insGACAGACTTT/insCCTACACGACGCTAATC
8	g.G6422T	Substitution
9	g.G6446GAATGA	insAATGA
10	g.T7258A	Substitution
11	g.T7672TTAAGGAAGGTGTAGAGTTTCTTA	insTAAGGAAGGTGTAGAGTTTCTTA
12	g.C8316CTTCTTTAAGTTTAGAATAG, CTACACACCCTCTTTTAAGAAAGG, CTGATCA	insTTCTTTAAGTTTAGAATAG/insTACACACCCTCTTTTAAGAAAGG/insTGATCA
13	g.A8378T	Substitution
14	g.A10888AGCTGATGTTACTAAAATAAAACCT, ATACAACTAGCTACAGAGAAG	insGCTGATGTTACTAAAATAAAACCT/insTACAACTAGCTACAGAGAAG
15	g.T10968TATATTGA	insATATTGA
16	g.T11187A	Substitution
17	g.T11880A	Substitution
18	g.C12420CTAGTTA	insTAGTTA
19	g.A12615AGCTGATGTTACTAAAATAAAACCT	insGCTGATGTTACTAAAATAAAACCT
20	g.C12713CAG, CACACCCTCTTTTAAGAAAG	insACACCCTCTTTTAAGAAAG
21	g.T13408A	Substitution

### Comparative ROC analysis of NMD variant burden

To evaluate the discriminative power of NMD-associated mutations for predicting severe COVID-19, two ROC analyses were conducted. The first model included all 21 NMD-associated nonsense mutations, while the second included only the 12 variants that demonstrated statistically significant associations with disease severity.

**Fig. 3:**
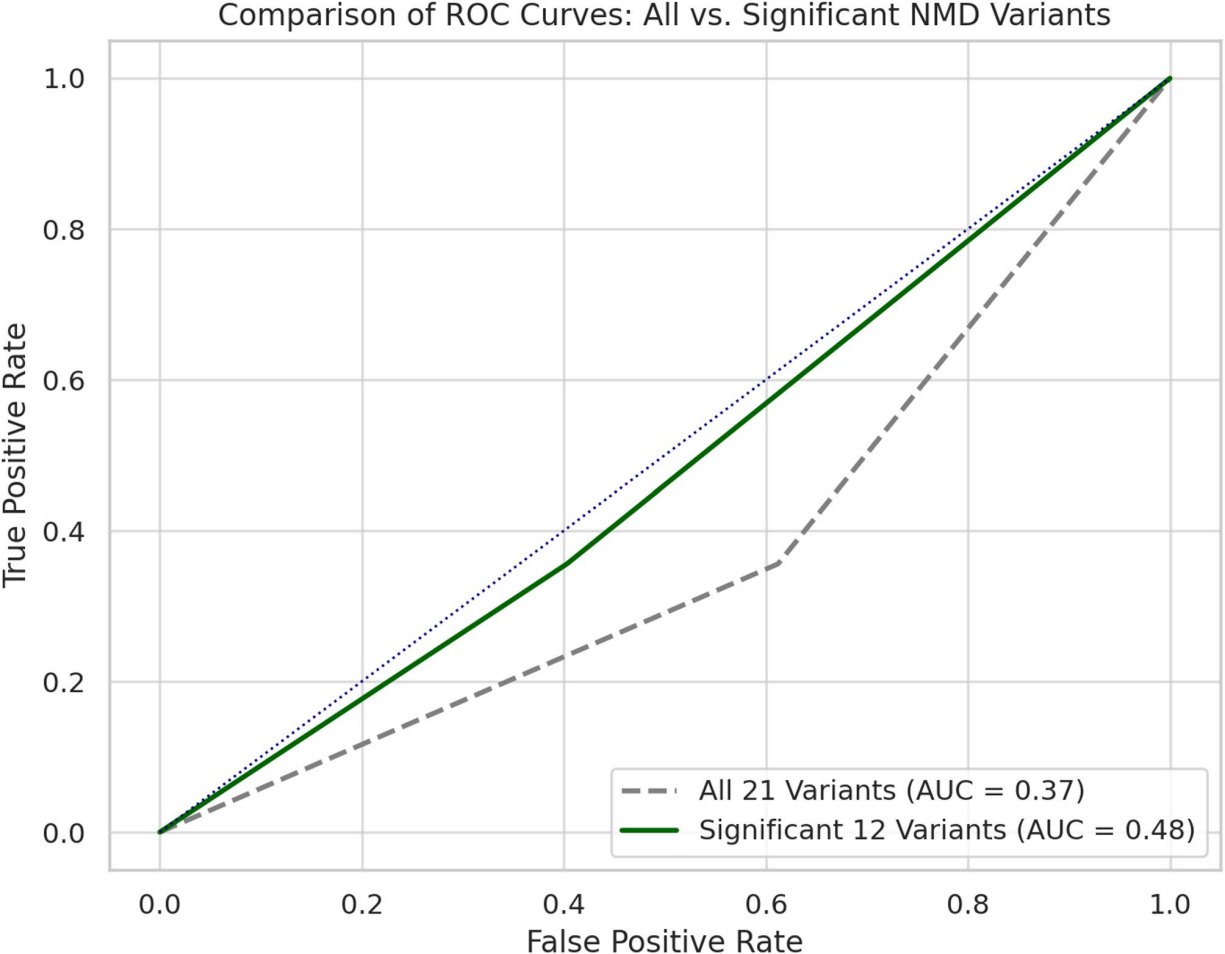
Comparison of ROC Curves for NMD Mutation Burden. ROC curves comparing the predictive value of two models for identifying severe COVID-19 cases based on NMD mutation burden. The grey dashed line represents the ROC curve using all 21 NMD-associated variants (AUC = 0.37). The solid green line represents the ROC curve using only the 12 statistically significant variants (AUC = 0.48). Both curves fall below the line of no discrimination (diagonal), indicating that NMD mutation burden alone, whether broadly defined or refined to significant variants, has limited predictive power for distinguishing severe from non-severe COVID-19 outcomes

The ROC curve using all 21 variants produced an AUC of 0.37, suggesting poor predictive ability. This may be due to the inclusion of variants with no functional relevance, which likely diluted the impact of protective variants. In contrast, the model restricted to the 12 significant variants yielded a slightly improved AUC of 0.48 but still failed to achieve acceptable discriminative performance (Fig. 3). These findings indicate that although certain NMD mutations are statistically associated with reduced severity, they do not function as effective standalone biomarkers for predicting disease outcomes. They may, however, contribute value as part of a multivariable model incorporating host factors and clinical data.

### Multivariable logistic regression analyses of NMD status, viral lineage, and severe COVID-19

A total of 129 patients were included, with WHO variant distribution as follows: Delta (*n* = 47, 36.7%), Omicron (*n* = 41, 32.0%), VOIs/VOC**s** (*n* = 31, 24.2%), and Alpha (*n* = 10, 7.0%). Severity rates varied by lineage: Alpha 11.1%, Delta 25.5%, Omicron 39.0%, and VOIs/VOCs 45.2%. The complete list of patient-level lineage assignments is provided in Supplementary Table S1.

#### Association between NMD-positive status and disease severity

In the host-adjusted model (age, sex, comorbidities), NMD-positive status was significantly associated with reduced odds of severe COVID-19 (adjusted OR = 0.34, 95% CI: 0.16–0.72, *p* = 0.005).

#### Association between viral lineage and disease severity

When WHO variant was modeled as the primary exposure (Delta as the reference), Omicron infection was associated with 1.91 times higher odds of severe disease compared to Delta, although this did not reach statistical significance (95% CI: 0.77–4.77, *p* = 0.164). Alpha and VOIs/VOCs could not be stably estimated due to small sample sizes and/or lack of variation in severity within the group.

#### Association between NMD status and disease severity, adjusted for viral lineage

When WHO variant was added to the NMD model (Delta reference), the association between NMD-positive status and severity was attenuated (adjusted OR = 0.67, 95% CI: 0.03–13.84, *p* = 0.793) and lost statistical significance. In this lineage-adjusted model, Omicron retained a similar effect size compared to the lineage-only model (adjusted OR = 1.87, 95% CI: 0.74–4.73, *p* = 0.184). The attenuation of the NMD association reflects that part of the observed severity variation was explained by differences in viral lineage. The full regression outputs for all three models are presented in Table 4.

Age showed a trend toward significance (*p* = 0.078), indicating a possible increase in risk with advancing age, though not reaching conventional significance. Comorbidities also demonstrated a borderline effect (*p* = 0.082), consistent with previous findings on increased risk of severe outcomes. Sex (male vs. female) was not significantly associated with severity in this model (*p* = 0.670).

These findings support the hypothesis that NMD-triggering mutations may attenuate the pathogenic potential of SARS-CoV-2 and reinforce the importance of incorporating viral genomic features in clinical severity prediction models.

**Table 4:** Multivariate logistic regression models predicting severe COVID-19 disease

Variable	NMD vs. Severity Model			Lineage vs. Severity Model			NMD vs. Severity, lineage-adjusted		
	OR	95% CI	*P*-value	OR	95% CI	*P*-value	OR	95% CI	*P*-value
Age	1.02	1.00–1.04	0.078	1.02	1.00–1.04	0.078	1.02	1.00–1.04	0.078
Sex (Male)	1.18	0.56–2.49	0.670	1.18	0.56–2.49	0.670	1.18	0.56–2.49	0.670
Comorbidities	1.94	0.92–4.09	0.082	1.94	0.92–4.09	0.082	1.94	0.92–4.09	0.082
NMD_Positive	0.34	0.16–0.72	0.005	—	—	—	0.67	0.03–13.84	0.793
Viral lineage (WHO variant)									
Alpha	—	—	—	NE	NE	NE	NE	NE	NE
Omicron	—	—	—	1.9	0.77–4.72	0.164	1.87	0.74–4.73	0.184
VOIs/VOCs	—	—	—	NE	NE	NE	NE	NE	NE

Multivariable logistic regression models evaluating the association between NMD-positive status, viral lineage, and severe COVID-19. Model 1: Association between NMD-positive status and severity, adjusted for age, sex, and comorbidities. Model 2: Association between WHO variant (Delta reference) and severity, adjusted for age, sex, and comorbidities. Model 3: Association between NMD-positive status and severity after adjustment for WHO variant (Delta reference) and host factors. NE: Not estimable due to no variation in NMD status or insufficient cell counts.

### Synergistic influence of NMD-Related mutations on infection severity

To assess the association between NMD-associated mutations and COVID-19 severity, Chi-square tests were conducted, followed by False Discovery Rate (FDR) correction to account for multiple hypothesis testing. Variants 5, 7, 5 + 7, 5 + 18, and 15 exhibited significant associations with disease severity, with p-values < 0.05, and these associations remained robust after FDR correction (FDR < 0.05). Specifically, Variants 5 and 15 demonstrated the strongest associations, with p-values of 0.0000 and 0.0001, respectively, and corresponding FDR values of 0.0000 and 0.0002. In contrast, Variant 18, with a p-value of 0.137, did not show a significant association after FDR correction (FDR = 0.137), indicating no strong evidence for its role in disease severity. These results suggest that, while several mutations are strongly associated with COVID-19 severity, others, such as Variant 18, require further investigation to confirm their potential relevance. The use of FDR adjustment ensures that the identified associations are robust and not due to random chance, addressing concerns about multiple comparisons (Table 5).

These findings indicate that specific NMD-related mutations, particularly Variants 5, 7, and 15, may function as independent protective markers. Moreover, their combined presence appears to exert a cumulative, synergistic effect, further reducing the risk of severe disease.

**Table 5:** Association between COVID-19 severity and NMD-Associated variants with high interdependency (ID ≥ 0.65)

Infection severity	Positivity	*N* (%)	Mild	Moderate	Severe	*P*-value
Variant 5	Negative	98(76.6)	7(36.8)	54(80.6)	37(88.1)	0.0001
	Positive	30(23.4)	12(63.2)	13(19.4)	5(11.9)	
Variant 7	Negative	103(80.5)	10(52.6)	54(80.6)	39(92.9)	0.001
	Positive	25(19.5)	9(47.4)	13(19.4)	3(7.1)	
Variant 5 + 7	Negative	108(84.4)	10(52.6)	58(86.6)	40(95.2)	0.0001
	Positive	20(15.6)	9(47.4)	9(13.4)	2(4.8)	
Variant 5	Negative	98(76.6)	7(36.8)	54(80.6)	37(88.1)	0.000
	Positive	30(23.4)	12(63.2)	13(19.4)	5(11.9)	
Variant 18	Negative	80(62.5)	8(42.1)	44(65.7)	28(66.7)	0.137
	Positive	48(37.5)	11(57.9)	23(34.4)	14(33.3)	
Variant 5 + 18	Negative	101(78.9)	9(47.4)	55(82.1)	37(88.1)	0.001
	Positive	27(21.1)	10(52.6)	12(17.9)	5(11.9)	
Variant 12	Negative	125(97.7)	17(98.5)	66(98.5)	42(100)	0.034
	Positive	3(2.3)	2(10.5)	1(1.5)	0(0)	
Variant 15	Negative	98(76.6)	16(84.2)	67(100)	42(100)	0.000
	Positive	30(23.4)	3(15.8)	0(0)	0(0)	
Variant 12 + 15	Negative	125(97.7)	17(98.5)	66(98.5)	42(100)	0.034
	Positive	3(2.3)	2(10.5)	1(1.5)	0(0)	

## Discussion

This study provides novel evidence that specific SARS-CoV-2 nonsense-mediated decay (NMD)-associated mutations are significantly associated with reduced COVID-19 severity. Through comprehensive whole-genome sequencing and targeted analysis of nonsense mutations predicted to activate the host NMD pathway, we identified a panel of 21 NMD-inducing mutations primarily within ORF1ab and examined their correlation with clinical outcomes. Our results demonstrate that a subset of these mutations, particularly when co-occurring in specific combinations, may confer a protective effect by attenuating viral virulence.

NMD is a highly conserved post-transcriptional surveillance mechanism that degrades mRNAs harboring premature termination codons (PTCs) [36], thereby preventing the production of truncated, potentially deleterious proteins [37]. While traditionally considered a housekeeping function in eukaryotic cells, emerging research has revealed its role in host-pathogen interactions [38]. Several viruses have evolved mechanisms to evade or suppress the NMD pathway to enhance their replication [9]. Conversely, some viral mutations may inadvertently trigger NMD, potentially reducing viral fitness and pathogenesis [7, 38].

In the context of SARS-CoV-2, the role of NMD-associated mutations in shaping viral behavior remains underexplored. A study by ***Karousis et al.***. (2021) [39]suggested that coronaviruses possess RNA structural features that may modulate susceptibility to NMD, particularly in subgenomic RNAs [40, 41]. However, this study did not focus on nonsense mutations nor link these features to clinical outcomes. Similarly, ***Bakhshandeh et al.***. (2021) noted that the SARS-CoV-2 genome contains sequences that could interact with RNA surveillance pathways but stopped short of evaluating the phenotypic consequences of PTC-generating mutations [7, 42]. In contrast, our study explicitly investigates the presence of nonsense mutations, predicts their likelihood of inducing NMD, and directly correlates them with clinical severity, marking a clear advancement in the understanding of virus-host RNA interactions in COVID-19.

Beyond SARS-CoV-2, NMD has been implicated in other viral infections. In HIV-1, NMD is known to degrade viral transcripts with PTCs, and suppression of NMD enhances viral replication [10, 43, 44]. Hepatitis C virus (HCV) similarly interacts with the NMD pathway, where disruption of host NMD enhances virus stability and pathogenicity [45]. These findings support the broader concept that host NMD functions as a molecular barrier against viral infection [10, 45]. The current study builds on this body of literature by providing clinical-genomic evidence of this relationship in a large cohort of COVID-19 patients.

Importantly, five NMD-associated variants (Variants 5, 6, 7, 9, and 15) showed highly significant associations with mild disease, while three others demonstrated moderate or mild statistical significance. Multivariate analysis confirmed that the presence of at least one NMD-inducing variant was an independent protective factor (OR = 0.34, *p* = 0.005) after adjusting for age, sex, and comorbidities. Notably, neither age nor comorbidities reached conventional statistical significance, further underscoring the impact of viral genomic features on clinical outcomes.

While this study focuses on NMD-associated mutations, it is important to acknowledge that clinical effects may also be influenced by loss-of-function mutations, such as those in ORF3b and ORF8, which modulate immune responses. NMD-induced mutations may lead to the degradation of mRNAs with premature termination codons, preventing the production of truncated proteins that could impact viral replication or host interactions [46].

ROC curve analysis illustrated the limitations of using NMD mutation presence as a standalone predictor, with AUC values of 0.37 (all 21 variants) and 0.48 (only significant variants). This indicates that while such mutations correlate with severity on a population level, they do not suffice as individual predictive markers. Nevertheless, they may contribute to composite risk scores when integrated with host and clinical variables. Given the sparse, binary nature of the mutation features and the limited number of variants analyzed, the ROC curve analysis highlights the challenges of using NMD mutation presence as a reliable predictor. The relatively low AUC values suggest that while these mutations correlate with severity at the population level, they are not strong enough to serve as standalone predictive markers. However, they may still hold value when integrated into composite risk models alongside host and clinical variables.

A key novel contribution of this study lies in the analysis of synergistic effects among interdependent variants (ID ≥ 0.65). We observed that combinations of specific NMD variants conferred greater protective effects than individual mutations alone. For instance, the co-presence of Variants 5 and 7 was associated with a dramatic reduction in severe cases (from 92.9 to 4.8%, *p* = 0.000). Similar synergistic trends were seen with combinations involving Variants 12, 15, and 18. To our knowledge, this is the first study to explore such inter-variant interactions and their impact on disease severity in the context of SARS-CoV-2.

Another strength is the real-world clinical relevance of the cohort, drawn from 129 hospitalized patients with confirmed SARS-CoV-2 infection over a 12-month period. This provides a diverse and temporally representative dataset, improving generalizability across different phases of the pandemic. Moreover, the use of a rigorous filtering pipeline (including coverage depth ≥ 100, high-confidence variant calling, and NMD prediction based on position relative to exon-exon junctions) enhances the reliability of the mutation annotations.

The multivariable analyses demonstrate that while NMD-positive status was initially associated with a reduced risk of severe COVID-19, this relationship was attenuated after adjustment for SARS-CoV-2 lineage, with Omicron showing higher (though nonsignificant) odds of severity compared to Delta. This finding aligns with reports that variant-specific differences, particularly for Omicron may influence disease outcomes through changes in transmissibility, immune escape, and virulence profiles [47, 48]. Several prior studies have noted that host genetic variants, including those affecting mRNA processing and decay pathways, can modulate COVID-19 severity [49], but our results suggest that the apparent effect of NMD-linked mutations must be interpreted in the context of circulating viral lineages. The attenuation observed after lineage adjustment mirrors findings from genomic epidemiology studies, which have shown that viral genetic background can act as a confounder when assessing host genetic effects [50]. Together, these data emphasize that integrated host–virus analyses are necessary to disentangle the contributions of human genomic factors from variant-specific pathogenicity. Future studies with balanced representation of NMD-positive and NMD-negative individuals within each lineage are needed to confirm whether the observed protective association is consistent across SARS-CoV-2 variants or is modified by viral genomic background.

However, several limitations should be acknowledged. Functional validation of NMD induction was based on bioinformatic predictions rather than experimental data. Host genetic variation in NMD efficiency, which may impact the effects of viral mutations, was not considered. Although interdependency analysis suggests co-evolution of protective variants, the underlying mechanisms require further investigation. Future studies with broader range of mutation types, including protein-coding and non-coding variants are needed to better elucidate the genetic underpinnings of COVID-19 severity.

## Conclusion

In conclusion, this study adds a new dimension to the understanding of COVID-19 pathogenesis by highlighting the protective role of NMD-triggering nonsense mutations in SARS-CoV-2. The identification of specific variants and synergistic combinations that reduce disease severity underscores the importance of viral genomic surveillance not only for tracking transmission and immune escape but also for understanding disease-modulating features. These findings may inform future research on antiviral targets and risk prediction models that incorporate viral genetic data alongside host and clinical markers. While these mutations are associated with milder outcomes, causality cannot be inferred without further experimental validation.

## Supplementary Information

Supplementary Material 1

## Data Availability

The complete genome sequences of SARS-CoV-2 generated in this study (*n*=129) have been deposited in the GISAID database (Global Initiative on Sharing All Influenza Data) under the accession numbers listed in **Supplementary Table 2**. These data are publicly available to support global efforts in monitoring viral evolution and facilitate ongoing scientific collaboration. The submission follows the GISAID data-sharing framework as described by Shu and McCauley in 2017 **.**.

## Abbreviations

NMD: Nonsense-mediated decay
PTCs: Premature termination codons
COVID-19: Coronavirus that cause pandemic COVID-19
ROC: Receiving operating characteristics
OR: Odd ratio
AUC: Area under the curve
ARDS: Acute respiratory distress syndrome
mRNA: Messenger RNA
RT-PCR: Real-time reverse transcription polymerase chain reaction
WHO: World Health Organization
NP: Nasopharyngeal
VTM: Viral transport medium
PPE: Personal protective equipment
BSL-2+: Biosafety Level 2 Plus
cDNA: Complementary DNA
ANOVA: Analysis of variances
CIs: Confidence intervals
ORF1ab: Open reading frame 1ab
