# Supplementary material for: Suppnonsense-mediated decay-linked mutations in SARS-CoV-2 and their association with COVID-19 disease severity
Source: BMC Infect Dis. 2025 Sep 16;25:1108. doi: 10.1186/s12879-025-11609-8 (PMC12439368; doi:10.1186/s12879-025-11609-8)
Supplement: Supplementary file 1 — Supplementary Material 1 [file 12879_2025_11609_MOESM1_ESM.docx]

**Supplementary Table 1. Pango lineage, and WHO variant of the studied cohort**

| Patient code | Pango code | WHO |
| --- | --- | --- |
| C001 | B.1.1.7 | Alpha |
| C002 | AY.112 | Delta |
| C003 | AY.112 | Delta |
| C004 | AY.112 | Delta |
| C005 | B.1.1.7 | Alpha |
| C006 | B.1.1.7 | Alpha |
| C007 | B.1.1.7 | Alpha |
| C008 | BA.1 | Omicron |
| C009 | C.36.3 | VOIs/VOCs |
| C010 | C.36.3 | VOIs/VOCs |
| C011 | C.36.3 | VOIs/VOCs |
| C012 | C.36.3 | VOIs/VOCs |
| C013 | B.1.617.2 | Delta |
| C014 | B.1.617.2 | Delta |
| C015 | B.1.617.2 | Delta |
| C016 | AY.32 | Delta |
| C017 | AY.32 | Delta |
| C018 | AY.32 | Delta |
| C019 | C.36.3 | VOIs/VOCs |
| C020 | C.36.3 | VOIs/VOCs |
| C021 | C.36.3 | VOIs/VOCs |
| C022 | C.36.3 | VOIs/VOCs |
| C023 | C.36.3 | VOIs/VOCs |
| C024 | C.36.3 | VOIs/VOCs |
| C025 | C.36.3 | VOIs/VOCs |
| C026 | C.36.3 | VOIs/VOCs |
| C027 | C.36.3 | VOIs/VOCs |
| C028 | C.36.3 | VOIs/VOCs |
| C029 | C.36.3 | VOIs/VOCs |
| C030 | C.36.3 | VOIs/VOCs |
| C031 | C.36.3 | VOIs/VOCs |
| C032 | C.36.3 | VOIs/VOCs |
| C033 | C.36.3 | VOIs/VOCs |
| C034 | C.36.3 | VOIs/VOCs |
| C035 | C.36.3 | VOIs/VOCs |
| C036 | C.36.3 | VOIs/VOCs |
| C037 | C.36 | VOIs/VOCs |
| C038 | C.36 | VOIs/VOCs |
| C039 | C.36 | VOIs/VOCs |
| C040 | C.36.3 | VOIs/VOCs |
| C041 | C.36.3 | VOIs/VOCs |
| C042 | C.36.3 | VOIs/VOCs |
| C043 | C.36.3 | VOIs/VOCs |
| C044 | C.36.3 | VOIs/VOCs |
| C045 | C.36.3 | VOIs/VOCs |
| C046 | B.1.1.7 | Alpha |
| C047 | B.1.1.7 | Alpha |
| C048 | B.1.1.7 | Alpha |
| C049 | B.1.1.7 | Alpha |
| C050 | B.1.1.7 | Alpha |
| C051 | B.1.1.7 | Alpha |
| C052 | B.1.617.2 | Delta |
| C053 | B.1.617.2 | Delta |
| C054 | B.1.617.2 | Delta |
| C055 | B.1.617.2 | Delta |
| C056 | B.1.617.2 | Delta |
| C057 | B.1.617.2 | Delta |
| C058 | AY.112 | Delta |
| C059 | AY.112 | Delta |
| C060 | AY.112 | Delta |
| C061 | AY.32 | Delta |
| C062 | AY.32 | Delta |
| C063 | AY.32 | Delta |
| C064 | AY.32 | Delta |
| C065 | AY.32 | Delta |
| C066 | AY.32 | Delta |
| C067 | B.1.617.2 | Delta |
| C068 | B.1.617.2 | Delta |
| C069 | B.1.617.2 | Delta |
| C070 | B.1.617.2 | Delta |
| C071 | B.1.617.2 | Delta |
| C072 | B.1.617.2 | Delta |
| C073 | AY.32 | Delta |
| C074 | AY.32 | Delta |
| C075 | AY.32 | Delta |
| C076 | AY.32 | Delta |
| C077 | AY.32 | Delta |
| C078 | AY.32 | Delta |
| C079 | AY.32 | Delta |
| C080 | B.1.617.2 | Delta |
| C081 | B.1.617.2 | Delta |
| C082 | B.1.617.2 | Delta |
| C083 | B.1.617.2 | Delta |
| C084 | B.1.617.2 | Delta |
| C085 | B.1.617.2 | Delta |
| C086 | B.1.617.2 | Delta |
| C087 | B.1.617.2 | Delta |
| C088 | B.1.617.2 | Delta |
| C089 | B.1.617.2 | Delta |
| C090 | BA.5.2.20 | Omicron |
| C091 | BA.5.2 | Omicron |
| C092 | BA.5.2 | Omicron |
| C093 | BA.5.2 | Omicron |
| C094 | BA.5.3.1 | Omicron |
| C095 | BA.5.2.1 | Omicron |
| C096 | BA.2 | Omicron |
| C097 | BA5.2 | Omicron |
| C098 | BA.5.2 | Omicron |
| C099 | BA.5.2.20 | Omicron |
| C100 | BA.5.2 | Omicron |
| C101 | BA.5.2 | Omicron |
| C102 | BA.5.2 | Omicron |
| C103 | BA.5.2 | Omicron |
| C104 | BA.5.2 | Omicron |
| C105 | BA.5.2 | Omicron |
| C106 | BA.5.3.1 | Omicron |
| C107 | BA2.5.6 | Omicron |
| C108 | BA.5.2.27 | Omicron |
| C109 | BA.5.2 | Omicron |
| C110 | BA.5.2 | Omicron |
| C111 | BA5.2 | Omicron |
| C112 | BA.5.2 | Omicron |
| C113 | BA.5.2 | Omicron |
| C114 | BA.5.56 | Omicron |
| C115 | BA.4 | Omicron |
| C116 | BA.5.2 | Omicron |
| C117 | BA.4 | Omicron |
| C118 | BA.5.2 | Omicron |
| C119 | BA.5.3.1 | Omicron |
| C120 | BA.5.2 | Omicron |
| C121 | BA.5.2 | Omicron |
| C122 | BA.2 | Omicron |
| C123 | BA.2 | Omicron |
| C124 | BA.2 | Omicron |
| C125 | BA.2.40.1 | Omicron |
| C126 | BA.2.5 | Omicron |
| C127 | BA.2 | Omicron |
| C128 | BA.2 | Omicron |
| C129 | BA.2 | Omicron |

**Supplementary Table 2. Accession ID for the 129 samples:**

| **Accession ID** | | | |
| --- | --- | --- | --- |
| **EPI_ISL_14593701** | **EPI_ISL_14594551** | **EPI_ISL_14593723** | **EPI_ISL_14594243** |
| **EPI_ISL_14594519** | **EPI_ISL_14594552** | **EPI_ISL_14593724** | **EPI_ISL_14593742** |
| **EPI_ISL_14594520** | **EPI_ISL_14594553** | **EPI_ISL_14593725** | **EPI_ISL_14594246** |
| **EPI_ISL_14594521** | **EPI_ISL_14594554** | **EPI_ISL_14593726** | **EPI_ISL_14594248** |
| **EPI_ISL_14594522** | **EPI_ISL_14594555** | **EPI_ISL_14593727** | **EPI_ISL_14593743** |
| **EPI_ISL_14594523** | **EPI_ISL_14594556** | **EPI_ISL_14593728** | **EPI_ISL_14594237** |
| **EPI_ISL_14594524** | **EPI_ISL_14594557** | **EPI_ISL_14593729** | **EPI_ISL_14594262** |
| **EPI_ISL_14594525** | **EPI_ISL_14594234** | **EPI_ISL_14593730** | **EPI_ISL_14593744** |
| **EPI_ISL_14594526** | **EPI_ISL_14593699** | **EPI_ISL_14593731** | **EPI_ISL_14594252** |
| **EPI_ISL_14594527** | **EPI_ISL_14593700** | **EPI_ISL_14593732** | **EPI_ISL_14594251** |
| **EPI_ISL_14594528** | **EPI_ISL_14593701** | **EPI_ISL_14593733** | **EPI_ISL_14594250** |
| **EPI_ISL_14594529** | **EPI_ISL_14593702** | **EPI_ISL_14593734** | **EPI_ISL_14594258** |
| **EPI_ISL_14594530** | **EPI_ISL_14593703** | **EPI_ISL_14594244** | **EPI_ISL_14594257** |
| **EPI_ISL_14594531** | **EPI_ISL_14593704** | **EPI_ISL_14593735** | **EPI_ISL_14593745** |
| **EPI_ISL_14594532** | **EPI_ISL_14593705** | **EPI_ISL_14594235** | **EPI_ISL_14594269** |
| **EPI_ISL_14594533** | **EPI_ISL_14593706** | **EPI_ISL_14594249** | **EPI_ISL_14594270** |
| **EPI_ISL_14594534** | **EPI_ISL_14593707** | **EPI_ISL_14593736** | **EPI_ISL_14594268** |
| **EPI_ISL_14594535** | **EPI_ISL_14593708** | **EPI_ISL_14594247** | **EPI_ISL_14594271** |
| **EPI_ISL_14594536** | **EPI_ISL_14593709** | **EPI_ISL_14594245** | **EPI_ISL_14594263** |
| **EPI_ISL_14594537** | **EPI_ISL_14593710** | **EPI_ISL_14593737** | **EPI_ISL_14594264** |
| **EPI_ISL_14594538** | **EPI_ISL_14593711** | **EPI_ISL_14594260** | **EPI_ISL_14594265** |
| **EPI_ISL_14594539** | **EPI_ISL_14593712** | **EPI_ISL_14594238** | **EPI_ISL_14594267** |
| **EPI_ISL_14594540** | **EPI_ISL_14593713** | **EPI_ISL_14593738** | **EPI_ISL_14594266** |
| **EPI_ISL_14594541** | **EPI_ISL_14593714** | **EPI_ISL_14594239** | **EPI_ISL_14594275** |
| **EPI_ISL_14594542** | **EPI_ISL_14593715** | **EPI_ISL_14594241** | **EPI_ISL_14594255** |
| **EPI_ISL_14594543** | **EPI_ISL_14593716** | **EPI_ISL_14593739** | **EPI_ISL_14594274** |
| **EPI_ISL_14594544** | **EPI_ISL_14593717** | **EPI_ISL_14594261** | **EPI_ISL_14594253** |
| **EPI_ISL_14594545** | **EPI_ISL_14593718** | **EPI_ISL_14594240** | **EPI_ISL_14594273** |
| **EPI_ISL_14594546** | **EPI_ISL_14593719** | **EPI_ISL_14593740** | **EPI_ISL_14594254** |
| **EPI_ISL_14594547** | **EPI_ISL_14593720** | **EPI_ISL_14594259** | **EPI_ISL_14594272** |
| **EPI_ISL_14594548** | **EPI_ISL_14594256** | **EPI_ISL_14594236** |  |
| **EPI_ISL_14594549** | **EPI_ISL_14593721** | **EPI_ISL_14593741** |  |
| **EPI_ISL_14594550** | **EPI_ISL_14593722** | **EPI_ISL_14594242** |  |
